# Phantom and clinical evaluation of bone SPECT/CT image reconstruction with xSPECT algorithm

**DOI:** 10.1186/s13550-020-00659-5

**Published:** 2020-06-29

**Authors:** Noriaki Miyaji, Kenta Miwa, Ayaka Tokiwa, Hajime Ichikawa, Takashi Terauchi, Mitsuru Koizumi, Masahisa Onoguchi

**Affiliations:** 1 Department of Nuclear Medicine, Cancer Institute Hospital of Japanese Foundation for Cancer Research, 3-8-31 Ariake, Koto-ku, Tokyo, 135-8550 Japan; 2 Department of Quantum Medical Technology, Institute of Medical Pharmaceutical and Health Sciences, Kanazawa University, 5-11-80 Kodatsuno, Kanazawa, Ishikawa 920-0942 Japan; 3 Department of Radiological Sciences, School of Health Science, International University of Health and Welfare, 2600-1 Kitakanemaru, Ohtawara, Tochigi, 324-8501 Japan; 4 Department of Radiology, Toyohashi Municipal Hospital, 50, Aza Hachiken Nishi, Aotake–Cho, Toyohashi, Aichi 441-8570 Japan

**Keywords:** xSPECT, Bone SPECT, OSCGM, Iteration number, Novel reconstruction

## Abstract

**Background:**

Two novel methods of image reconstruction, xSPECT Quant (xQ) and xSPECT Bone (xB), that use an ordered subset conjugate gradient minimizer (OSCGM) for SPECT/CT reconstruction have been proposed. The present study compares the performance characteristics of xQ, xB, and conventional Flash3D (F3D) reconstruction using images derived from phantoms and patients.

**Methods:**

A custom-designed body phantom for bone SPECT was scanned using a Symbia Intevo (Siemens Healthineers), and reconstructed xSPECT images were evaluated. The phantom experiments proceeded twice with different activity concentrations and sphere sizes. A phantom with 28-mm spheres containing a ^99m^Tc-background and tumor-to-normal bone ratios (TBR) of 1, 2, 4, and 10 were generated, and convergence property against various TBR was evaluated across 96 iterations. A phantom with four spheres (13-, 17-, 22-, and 28-mm diameters), containing a ^99m^Tc-background at TBR4, was also generated. The full width at half maximum of an imaged spinous process (10 mm), coefficients of variance (CV), contrast-to-noise ratio (CNR), and recovery coefficients (RC) were evaluated after reconstructing images of a spine using Flash 3D (F3D), xQ, and xB. We retrospectively analyzed images from 20 patients with suspected bone metastases (male, *n* = 13) which were acquired using [^99m^Tc]Tc-(H)MDP SPECT/CT, then CV and standardized uptake values (SUV) at the 4^th^ vertebral body (L4) were compared after xQ and xB reconstruction in a clinical setup.

**Results:**

Mean activity concentrations with various TBR converged according to increasing numbers of iterations. The spatial resolution of xB was considerably superior to xQ and F3D, and it approached almost the actual size regardless of the iteration numbers during reconstruction. The CV and RC were better for xQ and xB than for F3D. The CNR peaked at 24 iterations for xQ and 48 iterations for F3D and xB, respectively. The RC between xQ and xB significantly differed at lower numbers of iterations but were almost equivalent at higher numbers of iterations. The reconstructed xQ and xB images of the clinical patients showed a significant difference in the SUV_max_ and SUV_peak_.

**Conclusions:**

The reconstructed xQ and xB images were more accurate than those reconstructed conventionally using F3D. The xB for bone SPECT imaging offered essentially unchanged spatial resolution even when the numbers of iterations did not converge. The xB reconstruction further enhanced SPECT image quality using CT data. Our findings provide important information for understanding the performance characteristics of the novel xQ and xB algorithms.

## Background

Traditional bone imaging using ^99m^Tc-labeled phosphate compounds is widely applied as diagnostic tools for detecting osseous metastases and staging malignant disease [1–3]. Hybrid bone imaging using single-photon emission computed tomography/computed tomography (SPECT/CT) can enhance image quality due to attenuation correction (AC), scatter correction (SC), and precisely localized tracer uptake. Römer et al. showed that 92% of indeterminate lesions could be correctly classified by SPECT/CT with a pronounced benefit for bone lesions [4]. Utsunomiya et al. also reported significantly improved diagnostic confidence for fused SPECT/CT image datasets compared with side-by-side views of images using both SPECT and CT modalities [5]. Hybrid SPECT/CT imaging in three dimensions (3D) has overcome the problem of planar bone imaging, which has high sensitivity, but low specificity, and thus improved the accuracy of diagnosing bone lesions [6, 7].

Recent advances in SPECT technology have included not only hardware but also software, such as image reconstruction algorithms. Absolute quantitation of ^99m^Tc bone SPECT/CT is becoming feasible as a diagnostic tool and as a means of monitoring treatment effects [8, 9]. Previous phantom and clinical studies have found that the quantitative accuracy of SPECT imaging using ^99m^Tc is within ± 10% [10, 11]. A multicenter study of four SPECT/CT systems also found that quantitative accuracy was maintained within 10% using 3D iterative reconstruction with AC, SC, and resolution recovery [12]. However, more reliable quantitative data are needed before quantitative bone SPECT imaging could become a standard clinical diagnostic procedure. Currently, the need to develop novel SPECT imaging techniques associated with absolute SPECT quantitation has been discussed in terms of cost, standardized uptake values (SUV), and dosimetry [13–15]. Quantitative SPECT/CT can overcome the downsides of positron emission tomography and has thus contributed to the rapid spread of quantitative nuclear medicine applications [16, 17].

Improved spatial resolution of SPECT images helps to improve the quantitation, detection, and precise localization of small lesions [18]. However, the spatial resolution of SPECT images remains poor. Tsui et al. suggested that multimodal image reconstruction would remarkably improve SPECT image quality [19]. Kuwert et al. also focused on quantitation and multimodal reconstruction as a methodological advance to further increase the value of bone SPECT/CT imaging [13]. The impact of using multimodal reconstruction methodology in SPECT imaging should be better quantifiability and excellent diagnostic confidence, although this awaits validation.

Siemens® has introduced a technology called “xSPECT,” which includes a novel iterative image reconstruction algorithm (ordered subset conjugate gradient minimizer; OSCGM) based on conventional ordered subset expectation maximization (OSEM; Flash 3D; F3D) to improve multimodal alignment in image space and thus enhance image quality. Onoguchi et al. described the differences between OSEM and OSCGM algorithms in detail [20]. Briefly, the xSPECT technology applies the Mighell merit function to suppress noise caused by the fast convergence of OSCGM reconstruction. Additionally, the National Institute of Standards and Technology (NIST) traceable calibration ^57^Co point sources with 3% uncertainty (99% confidence level (CI)) were introduced by Siemens® to standardize quantitative ^99m^Tc-SPECT. The SPECT voxel counts based on accurate correction can be converted to activity concentrations (Bq/mL) using a system planar sensitivity correction factor measured with a ^57^Co source during reconstruction. This method of quantitative reconstruction is called “xSPECT Quant” (xQ). Siemens® also concurrently released bone-specific software with xSPECT features called “xSPECT Bone (xB)” [21], in which higher-resolution CT data were added to enhance reconstructed images at tissue boundaries. Therefore, xB produces images of tracer distribution with far better resolution than F3D [22]. Some clinical reports have described that xB bone SPECT images are more precise in terms of localization and offer better diagnostic confidence in staging malignant disease [23–25].

The fundamental theory of xB is that the application of image space information, divided into six tissue classes by higher-resolution CT data, minimizes interpolation errors in information obtained from anatomical modalities. Those reconstructed images have high spatial recognition due to denser spatial sampling. In contrast, xQ applies a CT-derived reconstruction mask to reduce background noise [26]. A comparison of the two reconstruction methods revealed unexpected behavior of xQ, which caused a decrease in the image quality of > 2 subsets [27, 28]. For both xQ and xB, developers also found that noise is lower, and resolution is higher in 3- than 6-degree sampling [29]. Quantitative and physical indexes such as recovery coefficients (RC), SUV, and noise characteristics typically depend on image reconstruction and the reconstruction parameters. Although xSPECT imaging also depends on different reconstruction parameters, its impact has not yet been clarified. The present study aimed to determine the performance characteristics of the novel xSPECT algorithm. To our knowledge, this is the first attempt to clarify the functional differences between xQ and xB based on phantom measurements and clinical data.

## Methods

### Data acquisition and reconstruction

All imaging data were acquired using a Symbia Intevo16 hybrid SPECT/CT system (Siemens Healthineers, Erlangen, Germany) comprising an integrated dual-head SPECT camera with a 16-slice helical CT scanner. We acquired SPECT images under the following parameters: ± 7.5% energy window at 140 keV with a lower scatter window of 15%, 3/8 " crystal thickness, low-energy high-resolution collimator, 256 × 256 matrix with 2.0-mm pixels, and a total of 120 projections of 15 s/view over 360° in a non-circular orbit continuous acquisition mode. Immediately following SPECT acquisition, CT images were acquired at 130 kV and 70 ref mA using adaptive dose modulation (CARE Dose 4D; Siemens Healthineers) with a 512 × 512 matrix, pitch 1.5, 0.8-s rotation, and 2 × 1.5-mm collimation. The CT data were reconstructed at a 3.0-mm slice thickness using a B31s attenuation filter (Siemens Healthineers).

We reconstructed the SPECT images using the algorithms F3D, xQ, and xB and a 6-mm 3D Gaussian filter with various combinations of one fixed subset and 1–96 iterations. The OSEM-based F3D is equipped with depth-dependent 3D resolution recovery using the Gaussian point-spread functions. The OSCGM-based xQ and xB are equipped with depth-dependent 3D resolution recovery using actual measured point-spread functions map over the entire FOV. The xB algorithm divides CT pixels into six tissue classes with smooth boundaries based on CT values or "zones" of air and lung, adipose, soft tissue, soft bone, cortical bone, metal material, and updates. The xB iterative operation can be weighted according to the corresponding zone class in the divided pixel; however, the iterative operation for each zone class based on the CT data does not increase the original count [21].

### Cross-calibration of SPECT imaging

Counts from SPECT images reconstructed with F3D and xSPECT were converted to activity concentrations based on a cross-calibration factor (CCF) obtained from the relationship between the reconstructed counts and activity concentrations as well as system planar sensitivity, for quantitative comparisons.

In SPECT images using F3D, a circular region of interest (ROI) to measure SPECT count density (counts/mL) was placed at the center of the cylindrical phantom on the central slice and at ± 1 and ± 2 slices from the center. The CCF was automatically calculated using GI-BONE software (Aze, Tokyo, Japan) as the ratio of the actual activity concentration (measured by the dose calibrator) in the phantom at the time of scanning to the measured SPECT count density per scan duration [30]. The dose calibrator used for cross-calibration was CRC-15R (final calibration date by manufacturer: April 19, 2005). The dose calibrator was also confirmed and calibrated with a site-specific NIST-traceable ^68^Ge/^68^Ga source every 3 months [31, 32] (final calibration date in site: December 18, 2019). Therefore, we assume that the uncertainty of the measurement by the dose calibrator is small. The actual SUV was calculated as:

$$ \mathrm{Calibration}\ \mathrm{factor}\times \mathrm{count}\ \mathrm{density}/\left(\frac{\mathrm{Injected}\ \mathrm{activity}}{\mathrm{Body}\ \mathrm{weight}\ \left(\mathrm{phantom}\ \mathrm{volume}\right)}\right) $$

Reconstruction with xQ and xB precisely determines images in units of becquerel/milliliter that are converted using system planar sensitivity with an NIST traceable ^57^Co source [21]. The system planar sensitivity is a necessary parameter to allow for conversion between the count rate and units of absolute activity. This is defined as a measure of how many counts the gamma camera detects for every unit of activity in its field of view. Therefore, system planar sensitivity was measured with the traceable point source without scattering and attenuation to realize accurate and reproducible quantitation [28, 33]. This source is recommended for all Siemens® users to improve SPECT quantitation. It was automatically converted to the quantitative SPECT/CT data by the VB10 software (Siemens Healthineers).

### Phantom studies

#### Phantom design

**Fig. 1:**
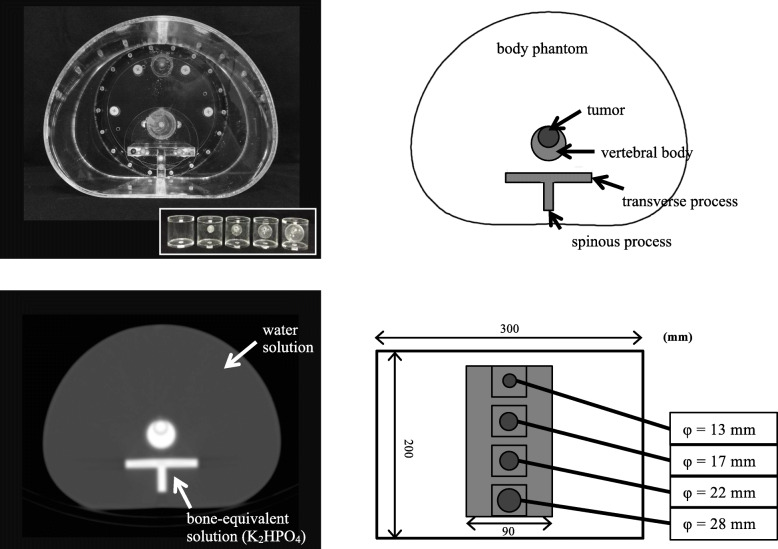
Custom-designed phantom configured with vertebral body, spinous and transverse process, and a sphere set inside the vertebral body to simulate bone metastasis

We custom-designed a physical three-dimensional phantom to determine the bone SPECT-specific distribution of activity and the linear attenuation coefficient (Fig. 1). This phantom can be used to generate SPECT images of bone metastasis with a realistic abdomen contour [34]. The phantom contains a ^99m^Tc solution to simulate soft tissue, the vertebral body, spinous and transverse process, and tumor region contained a bone-equivalent solution of K_2_HPO_4_ and ^99m^Tc [35]. The phantom experiments were conducted twice using different activity concentrations and sphere sizes as follows. Tumor, normal bone, and soft tissues in the phantom were immersed in a solution of ^99m^Tc. In the first round of experiments, a body phantom with four 28-mm diameter spheres was set and acquired at tumor-to-normal bone ratios (TBR) of 1, 2, 4, and 10 at a normal bone activity level of 50 kBq/mL. This phantom contained 8 kBq/mL of a ^99m^Tc solution as the background activity of the soft tissue. That is, the boundary and the background do not differ at TBR1, but the difference in the activity concentration increases as a function of a higher TBR. We determined the activity concentrations of the simulated soft tissue, normal bone, and tumor at 8, 50, and 200 kBq/mL (TBR4), respectively, in the second round of experiments using a phantom with 13-, 17-, 22-, 28-mm-diameter spheres.

#### Data analysis

The SPECT acquisition data in the first round of experiments were reconstructed using 1 subset and 1–96 iterations. We examined the effects of the reconstruction algorithms on various TBR in the 28-mm sphere and then determined the optimal reconstruction parameters based on the result of convergence characteristics. Phantom images containing simulated tumors of different sizes were continuously analyzed in terms of the spatial resolution of a 10-mm spinous process, the coefficient of variance (CV), the contrast-to-noise ratio (CNR) of the vertebral body, and RC as quantitative parameters. We drew profile curves on the spinous process and measured the full width at half maximum (FWHM). The CV was evaluated at an 80% circular ROI (ROI80%) placed at the center of the vertebral body. In addition, a total of sixty ROI80% including ± 1 and ± 2 slices were placed around the vertebral body to calculate as background CV. The CV was calculated as standard deviation (SD) divided by mean in the ROI. The CNR and RC at each sphere were determined by setting circular ROIs with diameters of 13, 17, 22, and 28 mm. The CNR at TBR 4 was calculated as (Hs − Hnb)/σnb, where Hs and Hnb are the activity concentrations measured in the spheres and normal bone, respectively, and σnb is the voxel SD in the normal bone. The RC was defined as the ratio of the SPECT-based and the true activity concentration (kBq/mL) for each sphere.

### Clinical study

#### Imaging protocol

We analyzed data from 20 consecutive patients who had undergone bone SPECT/CT imaging for metastatic prostate or breast cancer (male, *n* = 13; female, *n* = 7; median age, 62 years; range, 40–83 years; average weight, 65.2 ± 13.4 kg; range, 51.8–78.6 kg). The optimal parameters of the convergence characteristic in the phantom study were applied to the clinical reconstruction condition in xQ and xB. Bone SPECT/CT imaging proceeded from the abdomen to the pelvis ~ 2.5–4 h after delivering an intravenous injection of 1003.4 ± 102.8 MBq ^99m^Tc-methylene diphosphonate ([^99m^Tc]Tc-MDP; FUJIFILM Toyama Chemical, Tokyo, Japan) or hydroxymethylene diphosphonate ([^99m^Tc]Tc-HMDP; Nihon Medi-Physics, Tokyo, Japan). The average amount of injected ^99m^Tc was 15.9 ± 2.8 (range, 13.1–18.7) MBq/kg. The Ethics Committee at the Cancer Institute Hospital of JFCR approved this clinical study (approval no. 2015-1151). These clinical data were retrospectively analyzed, and the results did not influence any further therapeutic decision-making.

#### Data analysis

**Fig. 2:**
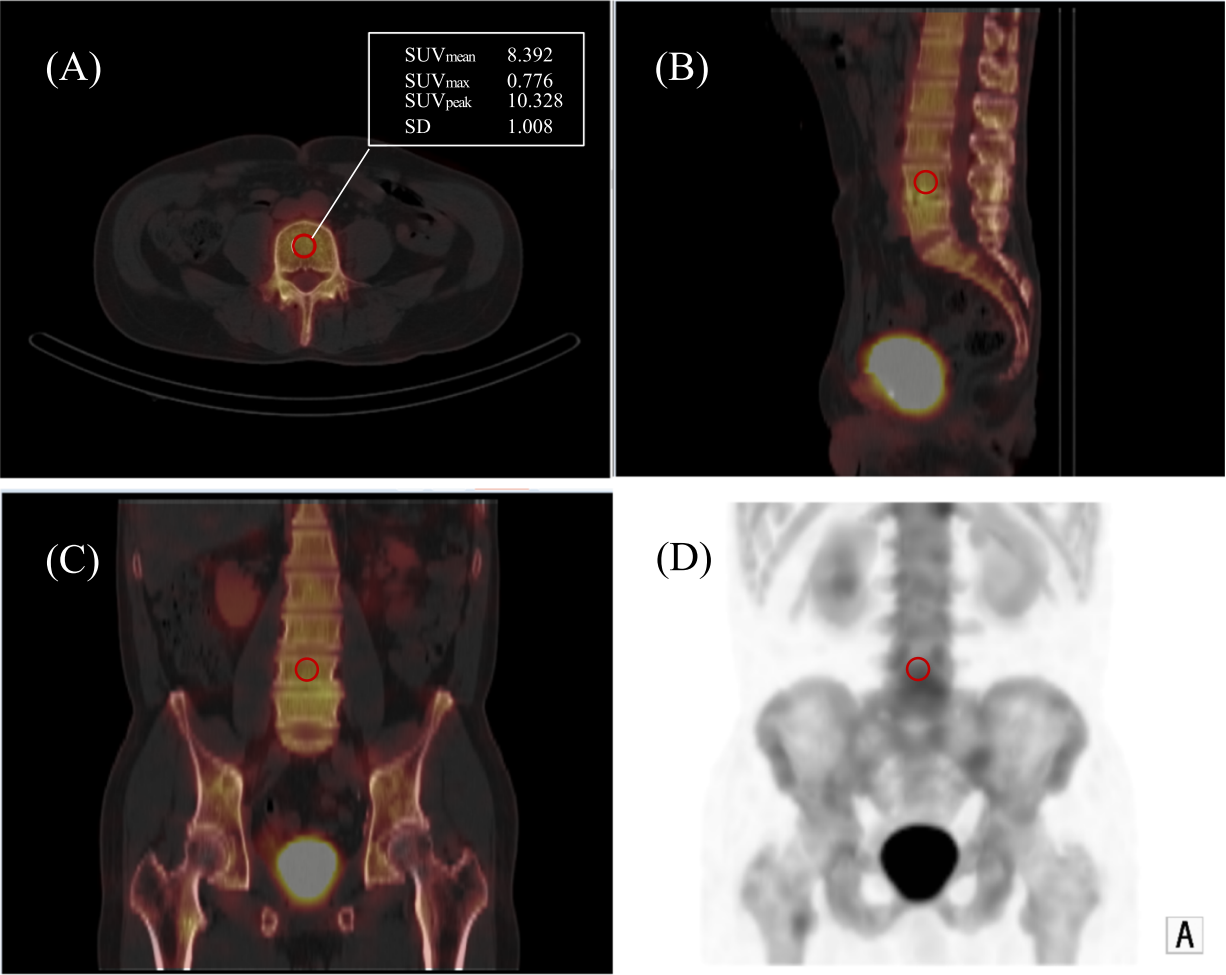
We set ROI80% (red circle) at the center of xB imaged based on a fused axial image, then adjusted by sagittal and coronal images. **a** Fused axial image. **b** Fused sagittal image. **c** Fused coronal image. **d** MIP image

The noise characteristics and quantitative performance of the clinical SPECT image were analyzed at the level of the 4th vertebral body (L4) [36]. We adjusted and placed a ROI of 80% size on the center of the axial slice in the section after measured the ROI of the vertebral body guided by the CT boundaries of the fused SPECT/CT images. We normalized the SUV_max_, SUV_mean_, and SUV_peak_ by the weight of each patient. An average and maximum concentration in milliliter within a ROI would produce an estimate of the SUV, which is defined here as SUV_mean_ and SUV_max_, respectively. SUV_peak_ has been suggested as an alternative to SUV_max_. SUV_peak_ within a setting ROI is an average SUV calculated within a fixed size (this is a sphere with a diameter of approximately 1.2 cm to require a 1-cm^3^ volume spheric ROI), placed highest uptake region including maximum pixel value. Because this VOI encompasses several pixels, SUV_peak_ is assumed to be less affected by image noise than SUV_max_. These data were analyzed using PETSTAT software (AdIn Research, Tokyo, Japan) (Fig. 2).

### Statistical analysis

All SUV and CV indices in the xQ and xB groups were compared using Wilcoxon signed-rank tests after evaluating the non-normal distribution using Kolmogorov-Smirnov tests. Values were considered statistically significant when *P* < 0.05. These data were statistically analyzed using SPSS Statistics software (IBM Corp., Armonk, NY, USA).

## Results

### Phantom studies

#### Convergence for various TBR

**Fig. 3:**
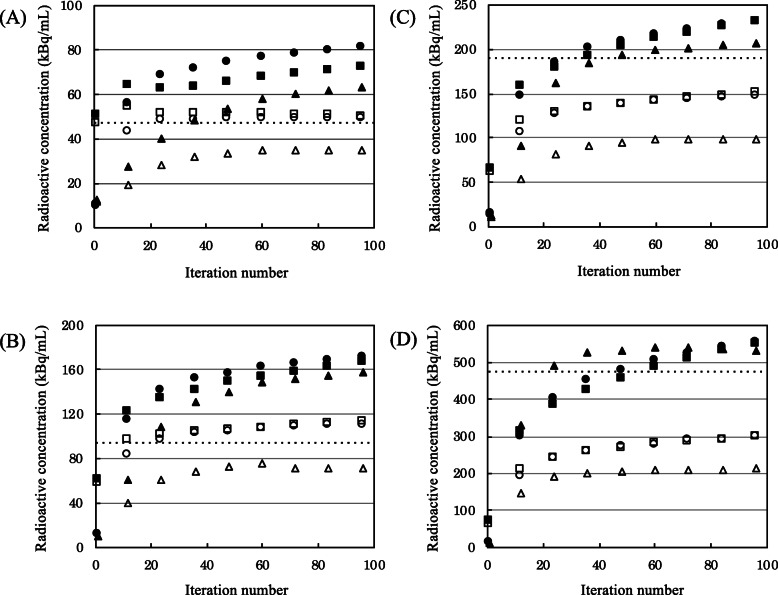
Reconstruction plots showing quantitative distribution in TBR1 (**a**), 2 (**b**), 4 (**c**), and 10 (**d**). The filled and unfilled symbols indicate maximum and mean activity concentrations, respectively. The dotted line is the actual activity concentration of phantom. Filled and unfilled triangles indicate Flash 3D (F3D). Filled and unfilled circles indicate xSPECT Quant (xQ). Filled and unfilled squares indicate xSPECT Bone (xB)

Figure 3 shows the SPECT data reconstructed using between 1 and 96 iterations. Regardless of the reconstruction model and iteration number, the means were better than the maximum activity concentrations for the two lowest TBR values (Fig. 3a, b), whereas those of the maximum activity concentrations were better results for the highest TBR values (Fig. 3c, d). In Fig. 3d, the maximum activity concentrations were the highest with F3D and better than those for both xQ and xB. On the other hand, the maximum activity concentration with xSPECT did not converge and increased in proportion to the iteration numbers. The mean activity concentration converged with increasing iterations regardless of the TBR. The mean activity concentrations of xQ and xB were essentially equivalent at > 24 iterations. The mean activity concentration was lower for F3D than xQ and xB.

#### Spatial resolution

**Fig. 4:**
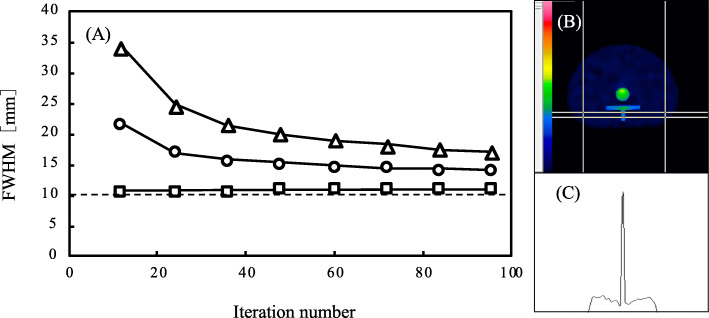
The FWHM measurement shown by the profile curve on the spinous process cross-section. **a** Spatial resolution of three reconstructions at various iterations. The dotted line is the actual size of the phantom. Unfilled triangle indicates Flash 3D (F3D). Unfilled circle indicates xSPECT Quant (xQ). Unfilled square indicates xSPECT bone (xB). **b** A sample measurement of an xB image at 12 iteration numbers. **c** A measurement profile of an xB image at 12 iteration numbers. The plateau signal around the spinous process is a distribution of background region in the phantom

**Fig. 5:**
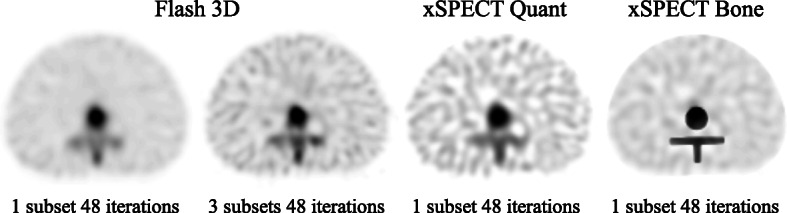
Representative transaxial images of SPECT datasets including three reconstructions at TBR4. Reconstructed images showed the xSPECT and F3D images with 1 subset and with 3 subsets at 48 iterations, respectively

Figure 4 shows the spatial resolution of the spinous process for various iterations. The FWHM with xQ and F3D considerably improved when the iteration number increased, but the spatial resolution produced by the xB algorithm was optimal. The FWHM of the xQ and F3D reconstructions converged at about 15 and 20 mm, respectively. In contrast, the xB values remained similar to the actual size (10 mm) regardless of iteration numbers. Figure 5 shows the results of the xSPECT and F3D images with 1 subset and F3D images with 3 subsets at 48 iterations, respectively. The boundary of the vertebral body was visually indistinct on reconstructed F3D and xQ bone SPECT images, whereas it was clearly visible in the reconstructed xB images. In terms of background region, xB and F3D images with 1 subset produced clearer images than xQ. Also, the xQ with 1 subset and F3D with 3 subsets respectively were visually equivalent level.

#### Noise characteristics

**Fig. 6:**
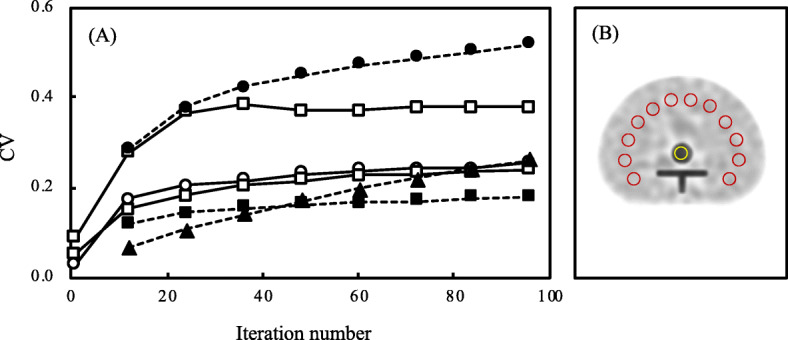
The coefficient of variance (CV) measurement in the ROI placed at the center of the vertebral body. **a** The CV as a function of iteration numbers. The black line is the CV of the vertebral body and the dot line in black is the background CV, respectively. Filled and unfilled triangles indicate Flash 3D (F3D). Filled and unfilled circles indicate xSPECT Quant (xQ). Filled and unfilled square xSPECT Bone (xB). **b** A sample measurement of an xB image at 1 subset and 48 iterations

**Fig. 7:**
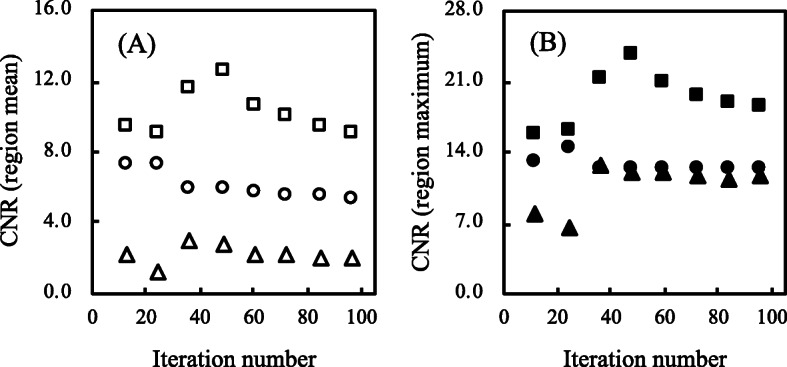
The contrast-to-noise ratio (CNR) measured by activity concentrations for the hot spheres and normal bone at TBR 4. **a** The mean CNR as a function of iteration numbers. **b** The maximum CNR as a function of iteration numbers. Filled and unfilled triangles indicate Flash 3D (F3D). Filled and unfilled circles indicate xSPECT Quant (xQ). Filled and unfilled square indicate xSPECT Bone (xB)

Figure 6 shows the CV of the vertebral body and background region according to the number of iterations, respectively. The CV in the vertebral body was higher in F3D than in xQ and xB as the iteration numbers increased, and the amount of noise was similar between xQ and xB. Those of xQ and xB at > 24 iterations were both relatively stable at 0.2. On the other hand, the background CV of xQ significantly was inferior to other reconstruction. The CV of xB and F3D showed an equivalent value at 48 iteration numbers. Figure 7 shows the CNR in the vertebral body region according to the iteration numbers. The mean and max CNR were similar for each reconstruction. Although the mean CNR was better in the order of xB, xQ, and F3D as the iteration numbers increased, the CNR of F3D and xB at > 48, and xQ at > 24 iterations decreased.

#### Recovery coefficient

**Fig. 8:**
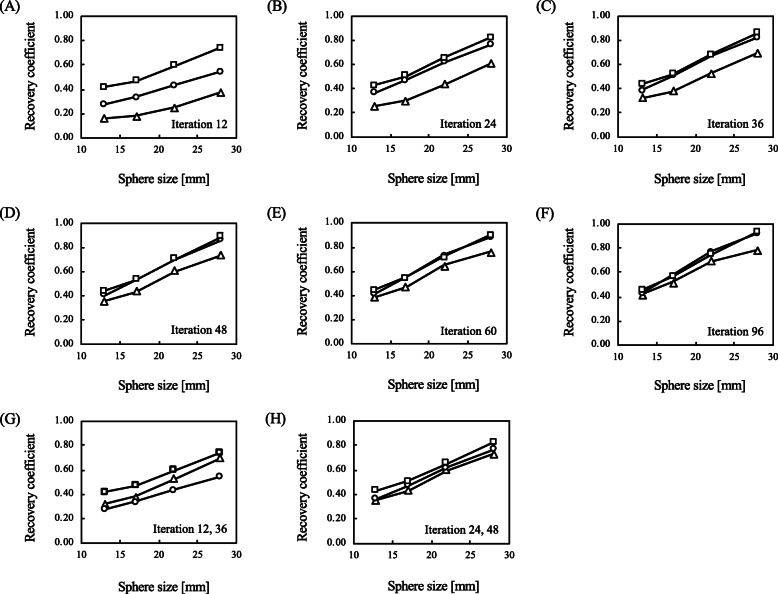
Recovery coefficients of three reconstructions at various numbers of iterations. The numbers of iterations in **a**, **b**, **c**, **d**, **e**, and **f** are 12, 24, 36, 48, 60, and 96, respectively. In addition, the numbers of iterations in **g** and **h** show the different parameters of 36 and 48 in Flash 3D and 12 and 24 in xSPECT, respectively. Unfilled triangle indicates Flash 3D. Unfilled circle indicates xSPECT Quant. Unfilled square indicates xSPECT Bone

Figure 8 shows the RC of the vertebral body for 12–96 iterations. The RC in all algorithms improved with increasing sphere size. The RC was relatively higher with xB than with the other algorithms at 12 iterations, and the differences in the RC between xQ and xB were essentially equivalent as a function of the increasing numbers of iterations. The RC was lower for F3D than xQ and xB at the same number of iterations, but the RC of F3D after 36 iterations was better than that of xQ after 12 (Fig. 8g).

### Clinical study

Table 1 shows the SUV_max_, SUV_peak_, SUV_mean_, and CV under clinical conditions. The quantitative SPECT values were much higher for some patients. The statistical findings showed a significant difference in the SUV_max_ and SUV_peak_ between xQ and xB. However, SUV_mean_ and CV on SPECT images reconstructed with xQ and xB did not significantly differ (*P* > 0.05).

**Table 1:** Values for SUV_mean_, SUV_max_, SUV_peak_, and CV of the 4th vertebral body in xQ and xB reconstructions

Quantitative indices	Reconstruction type	Mean ± SD	Min–max	*P* value
SUV_mean_	xQ	7.10 ± 5.81	2.50–30.34	0.084
	xB	7.03 ± 5.91	2.29–30.76	
SUV_max_	xQ	12.29 ± 11.84	4.87–58.16	0.021
	xB	11.90 ± 12.16	4.67–59.07	
SUV_peak_	xQ	11.63 ± 11.25	4.68–55.73	0.001
	xB	11.24 ± 11.37	4.56–55.52	
CV	xQ	0.28 ± 0.17	0.13–0.80	0.141
	xB	0.26 ± 0.17	0.12–0.69	

## Discussion

We validated novel xSPECT and conventional F3D reconstruction algorithms using experimental data derived from phantoms. Differences between xB and xQ were quantified based on clinical data from patients. The phantom study found image quality and quantitative accuracy of xSPECT were considerably superior to those of F3D. However, background noise obviously differed visually for xQ without weighted correction compared with F3D due to increasing noise caused by fast convergence. We also found that the high spatial resolution of xB was maintained regardless of the iteration numbers. The SUV_max_ and the SUV_peak_ in the clinical study significantly differed between xQ and xB; thus, we concluded that xB could serve as an essential diagnostic tool for bone SPECT imaging in terms of quantitative accuracy and spatial resolution.

Regardless of the reconstruction models, the maximum activity concentration in the TBR1 and TBR2 spheres was overestimated compared with actual activity concentration (Fig. 3). This can be explained by the fact that the maximum activity concentration in the sphere theoretically increased because of increasing statistical noise at lower counts [37]. The activity is pushed into the contours of the sphere due to the spatial constraint by a CT-based intensity masking for OSCGM. Tran-Gia et al. showed the mean xQ inside the sphere remained relatively similar to F3D; the distribution in the profile curve was drastically changed compared to F3D. The maximum in the sphere center was increasing, while the edge was decreasing [27]. Therefore, no convergence was reached for the SUV_max_ of xSPECT in Fig. 3, and the recovery of xSPECT was highly dependent on the iteration number. In contrast, F3D for higher TBRs is independent of the total iteration numbers; the maximum activity concentration of the F3D exceeded those of xSPECT (Fig. 3d). The merit function incorporated in xSPECT might enhance noise suppression as a function of higher activity concentration. However, this effect reduced for higher iteration numbers because xSPECT does not converge. The xSPECT reconstruction has several unknown features, so this is only one potential explanation. On the other side, the mean activity concentration approached the actual activity concentration at lower TBR. When the activity concentrations of tumor and normal bone were equal (TBR = 1), spill-out by partial volume did not occur because the activity concentrations inside and outside the ROI were almost equivalent. TBR 1 was slightly overestimated due to the activity concentration being increased by the statistical noise. At a higher TBR, the mean activity concentration was underestimated due to spillage from the sphere into the background [38]. The quantitative differences between F3D and xSPECT are influenced by statistical noise based on convergence and by partial volume effects caused by lower spatial resolution. Our results showed that the mean activity concentrations for F3D essentially converged within 48 iterations, but those for xQ and xB similarly converged at > 24 iterations. The FWHM for the xQ after 36 iterations converged, and the RC between the xB and xQ was almost equivalent at over 36 iterations. The maximum activity concentrations with xSPECT did not converge even for high iteration numbers as shown in Fig. 3. The iteration numbers are associated with a trade-off between signal and noise. Considering the increase in noise, we determined that 30 iterations were the most appropriate for xSPECT reconstruction in clinical practice.

The FWHM of F3D after > 36 iterations was better than xQ after 12 iterations that have not fully converged; however, the FWHM with xQ and F3D considerably improved and fully converged at ~ 15 and 20 mm, respectively, at a high number of iterations. Therefore, image quality was better for xQ than F3D at the appropriate parameter. In contrast, the xB algorithm divided into zone class generated unique results, unlike the observed xQ. The spatial resolution for xB remained almost unchanged even for lower iteration numbers, and the actual size of 10 mm was almost achieved. The zone class of each tissue was based on high-resolution CT images with delineated edges; therefore, the FWHM of xB reflects the relationship to CT resolution. Additionally, the xB iterative operation is weighted by zero or other value according to the corresponding zone class in the divided pixel [21]. We considered that not only bone classes weighted by the optimal value, but also non-bone classes weighted by zero with a zonal map were responsible for the improved spatial resolution using the xB technology.

The xSPECT can compensate for SPECT images by applying the merit function in the higher noise caused by the faster convergence of OSCGM reconstruction. This method of reconstruction adopts the Mighell-modified chi-squared gamma statistic algorithm. Shinohara et al. indicated that Mighell-modified noise suppression was better than other image reconstructions based on chi-square statistics [39]. The CV of xQ and xB did not exceed that of F3D at 12 iterations regardless of the iteration numbers. Thus, xSPECT with the Mighell-modified merit function considerably suppressed noise compared with F3D algorithms at the same number of iterations. For one subset of reconstructed images, a more apparent problem is the increasing image noise in the background region and in hot spheres according to the iteration numbers. The background noise in the xQ image rapidly increased and significantly was inferior to the other reconstructed images at 48 iterations (Fig. 5 and Fig. 6). However, Armstrong et al. reported that the greatest CNR for xQ is achieved at 48 iterations for one subset [28]. Our findings indicated that the greatest CNR for xQ was at 24 iterations and that the RC was higher than for F3D at 48 iterations (Figs. 7 and 8h). On the other hand, xB suppressed image noise more effectively than F3D and xQ (Fig. 6). Because xB reconstruction has weighted correction for every zone class, the impact of noise suppression differed between xQ and xB [40]. The CNR in xB reached the maximum at 48 iterations, and noise suppression decreased in xB at > 48 iterations. In regions with inadequate uptake such as soft tissues, the xQ based on the OSCGM algorithm might lead not only to an increased CV according to iteration setting, but also to ramifications for lesion detectability. Therefore, the xQ requires further careful optimization of the iteration numbers than F3D and xB.

The present study assessed data from 20 patients with suspected bone metastases. The SPECT values of measured L4 had a wide SUV range because some patients had various pathologies (bone metastasis (*n* = 7), degenerative (*n* = 5), and trauma (*n* = 3)). Our clinical study found a significant difference in SUV_peak_ and SUV_max_, and this quantitative difference between xQ and xB could be interpreted as noise suppression owing to weighted correction based on zone map system. Because the SUV_peak_ is less susceptible to statistical noise compared with SUV_max_ [37], it significantly differed between xQ and xB (*p* = 0.001). The clinical xB image with high resolution can not only reveal bone microlesions but also improve diagnostic confidence [23]. Therefore, we considered that clinical evaluation for xB images with SUV_peak_ could provide more accurate and reliable diagnostics. To calculate SUV_peak_ entails expressing the maximum average voxel value within a spherical volume of 1 cm^3^, but the xB is useful to enhance diagnostics for bone SPECT images in terms of quantitative and qualitative superiority. However, the quantitative variation caused by misalignments such as motion and respiratory errors during clinical scanning is a concern. Reconstruction using the xB algorithm might behave differently due to the unique zone map system. Therefore, misalignment between SPECT and CT images due to respiratory errors such as those caused by the ribs and sternum should be considered when clinically applying xB.

The present study has several limitations. The reconstructed SPECT images were assessed using different cross-calibration methods. The CCF on quantitative SPECT images varied depending on the activity concentration [41]. Thus, slight quantitative errors might arise between the F3D and xSPECT models. In addition, the body type of the 20 patients and the amounts of injected tracer were essentially standard (average, 15.9 ± 2.8 MBq/kg). We could not consider dependence on physique into consideration, and the effects of factors such as counts and scattering remain unclear. Further study is required to assess the relationship between body type and the quality of images reconstructed using the xSPECT algorithm.

## Conclusions

Bone images were qualitatively and quantitatively improved when reconstructed using OSCGM-based xSPECT (xQ and xB) compared with the OSEM-based F3D reconstruction. The quality of images under optimized xB reconstruction conditions was better because of sharper demarcation and lower background noise. One unique aspect of the bone structures in xB reconstructions is that the image content such as spatial resolution was independent of the iteration numbers. Our findings provide important information that should facilitate understanding of the performance characteristics of the novel xQ and xB algorithms.

## Data Availability

All data generated or analyzed during this study are included in this published article.

## Abbreviations

xQ: xSPECT Quant
xB: xSPECT Bone
OSCGM: Ordered subset conjugate gradient minimizer
F3D: Flash 3D
FWHM: Full width at half maximum
CV: Coefficients of variance
RC: Recovery coefficients
SUV: Standardized uptake value
L4: 4th vertebral body
AC: Attenuation correction
SC: Scatter correction
SPECT/CT: Single-photon emission computed tomography/computed tomography
OSEM: Ordered subset expectation maximization
CCF: Cross-calibration factor
TBR: Tumor-to-normal bone ratios
ROI: Regions of interest
